# Trajectories of Single- or Multiple-Substance Use in a Population Representative Sample of Adolescents: Association with Substance-Related and Psychosocial Problems at Age 17

**DOI:** 10.3390/brainsci15040331

**Published:** 2025-03-22

**Authors:** Rene Carbonneau, Frank Vitaro, Mara Brendgen, Michel Boivin, Sylvana M. Côté, Richard E. Tremblay

**Affiliations:** 1 Department of Pediatrics, Faculty of Medicine, University of Montreal, 3050 Edouard-Montpetit, Suite 225, Montréal, QC H3T 1J7, Canada; 2 Sainte-Justine Hospital Research Center, Montréal, QC H3T 1C5, Canada; 3 Research Unit on Children’s Psychosocial Maladjustment, University of Montreal, Montreal, QC H3T 1J4, Canada; 4 School of Psychoeducation, University of Montreal, Montréal, QC H3T 1J7, Canada; 5 Department of Psychology, University of Quebec in Montreal, Montréal, QC H2L 2C4, Canada; 6 Department of Psychology, Laval University, Quebec, QC G1V 0A6, Canada; 7 Department of Social and Preventive Medecine, School of Public Health, University of Montreal, Montréal, QC H3T 1J7, Canada

**Keywords:** substance use, polysubstance use, adolescence, trajectories, preadolescence, risk factors, development

## Abstract

Background: Research is limited regarding the relationship between adolescent substance use and polysubstance use (SU/PSU) as well as their outcomes later in adolescence, while accounting for early risk factors. This study explored substance-related and psychosocial outcomes at age 17 associated with SU/PSU developmental trajectories in a population-representative cohort from Quebec, Canada (N = 1593; 48.4% male), while controlling for preadolescent individual, familial, and social risk factors. SU/PSU included concurrent use of alcohol (AL), cannabis (CA), and other illicit drugs (ODs). Methods: Self-reported substance use data were collected at ages 12, 13, 15, and 17. Latent growth modeling identified five trajectories: Non-Users (12.8%) and four SU/PSU classes (5.8–37.5%) with varying severity based on onset, frequency, and substance type. Multinomial regression, using non-users as the reference group, assessed trajectory associations with age-17 outcomes. Individual, familial, and social risk factors assessed at ages 10–12 served as control variables. Results: Adolescents in high-risk SU/PSU classes showed the most negative substance-related and psychosocial outcomes compared to non-users and lower-risk SU/PSU classes. Lower-risk SU/PSU classes showed higher maladjustment than non-users. Conclusions: The findings highlight a dose–response relationship between SU/PSU trajectories and late-adolescent outcomes, independent of preadolescent risk factors. Results emphasize the importance of longitudinal studies that incorporate multiple substances to better capture the complexity of teenagers’ involvement in substance use throughout adolescence.

## 1. Introduction

Substance use (SU) has become a significant public health issue among adolescents in recent decades [1]. Despite ongoing prevention initiatives [2] and observed shifts in the types and frequencies of adolescent drug use, substance-related problems continue to be a major concern [3,4]. One growing issue is polysubstance use (PSU), the concurrent use of multiple substances [5], which has been increasingly highlighted in recent population surveys [6,7]. PSU is associated with higher overall substance consumption and a greater likelihood of developing substance use disorders (SUDs) [3,6], both of which have been linked to potential negative effects on brain development [8,9]. Furthermore, adolescent SU is associated with a range of psychosocial difficulties including anxiety, depression, conduct problems, and delinquency [10,11,12,13,14]. Additionally, more than half of individuals who develop substance use disorders in adulthood initiate SU during adolescence, underscoring the importance of early intervention [15]. However, there remain significant gaps in our understanding of SU and PSU in adolescents that may hamper our ability to intervene effectively.

First, there is a scarcity of research directly comparing single-substance users with polysubstance users. Most studies compared substance users to non-users [3,8], which limits the ability to isolate the specific factors that contribute to the more severe outcomes observed in PSU. It remains unclear whether these outcomes are primarily due to the increased frequency of use associated with PSU, the combination of substances used, or both. Second, much of the existing research on adolescent SU and PSU has relied on cross-sectional data [3]. Since many adolescents experiment with substances at some point, longitudinal studies that track substance use over time are necessary to more accurately assess the course of SU and PSU, the types of substances used, and the frequency of use [8,16]. Third, most research has focused on clinical or at-risk samples to establish the developmental trajectories of SU and PSU across adolescence [3,17]. Population-based studies are essential to better understand the broader developmental processes that contribute to SU and its consequences, including the identification of risk factors associated with the onset and progression of PSU in the general adolescent population [18]. Fourth, few studies have addressed the relation between PSU and later outcomes while accounting for the influence of earlier risk factors. From a developmental perspective, this is an important gap, as SU and PSU are often part of broader developmental pathways triggered by individual and environmental risk factors such as male sex, externalizing and internalizing disorders, family adversity, lower socioeconomic status (SES), inadequate parenting, and association with deviant peers [19,20,21]. These factors are known to be linked to both substance-related and psychosocial outcomes [22,23], complicating the interpretation of PSU’s role in later outcomes.

To better understand the consequences of SU and PSU on adolescents’ psychosocial adjustment, a longitudinal study is needed that examines both single-substance and polysubstance use patterns using repeated measures from a population sample. Such a study should also consider earlier risk factors that contribute to both types of SU. This approach would allow researchers to determine whether adolescent SU and PSU independently contribute to psychosocial maladjustment, or whether different developmental patterns of use lead to distinct forms of maladjustment. Understanding these dynamics would provide valuable insights for designing interventions tailored to the specific needs associated with different patterns of adolescent SU and PSU, as well as their continuity into later SU or other psychosocial issues [24]. A comprehensive, developmental approach that follows the continuum of risk factors, SU patterns, and psychosocial outcomes is therefore critical for advancing our understanding and improving intervention strategies.

### The Present Study

The aim of this study was to investigate age-17 outcomes associated with different developmental patterns of adolescent SU/PSU across the ages of 12, 13, 15, and 17 years in a population-representative cohort, controlling for preadolescent risk factors. To this end, we capitalized on the longitudinal patterns describing the co-occurring use of alcohol (AL), cannabis (CA), and other illicit drugs (ODs)—as well as on their association with preadolescent individual, familial, and social risk factors—identified in a previous study with this sample [11]. Using latent growth modeling, that study identified a class of non/low users (N = 204, 12.8%) and four distinct classes of adolescent SU/PSU patterns (N = 93–597; 5.8–37.5%), which varied in age of onset, frequency, and the type of substances used, and were differentially associated with preadolescent risk factors (see Methods and Results sections for a detailed description).

The present study was thus concerned with the differential adjustment of adolescents aged 17 following different patterns of SU/PSU compared to non/low users, as well as to each other. More specifically, this study examined the extent to which these patterns of use predicted substance-related issues (i.e., daily smoking, substance abuse, variety of substances used, and problems related to SU) in late adolescence. We also examined other indices of psychosocial maladjustment outcomes typically associated with SU or PSU (i.e., anxiety, depression, or conduct problems symptoms, and problems with the justice system). These associations were examined while controlling their preadolescent individual (male sex, internalizing and externalizing problems), familial (family adversity and appropriate parenting), and social (SES and associating with deviant peers) risk factors identified previously [11]

Our hypotheses were informed by prior studies documenting similar outcomes [3,6,10,11,12,13,14] and increasing evidence linking adolescent SU to brain development [8,9]. First, we expected that adolescents engaging in high-risk PSU would exhibit the most detrimental outcomes, particularly regarding substance-related indices, compared to low or non-users and those with lower-risk SU/PSU patterns. Second, acknowledging that lower-risk SU/PSU is also associated with negative outcomes relative to low or non-use [12,13,14], adolescents with such lower-risk use patterns should demonstrate poorer adjustment than low or non-users, but to a lesser extent than those engaging in higher-risk PSU.

## 2. Materials and Methods

### 2.1. Participants

Participants in this study were drawn from a birth cohort of 2226 infants (92% of White European ancestry, 48.4% male) from the Quebec Longitudinal Study of Child Development (QLSCD), a population-representative sample of children born in the Canadian province of Quebec between October 1997 and July 1998 [25]. Eligibility for the study was restricted to mothers who gave birth between 24 and 42 weeks of gestation and who spoke either English or French (Canada’s official languages; French is the primary language for 80% of Quebec’s population). Initial assessments of children and their families were conducted when the children were approximately 5 months old. A comprehensive description of the QLSCD methodology from inception and across assessment times can be found elsewhere [25].

The trajectories of SU/PSU were based on adolescents’ self-reported alcohol use (AL), cannabis use (CA), and other drug use (OD) at ages 12, 13, 15, and 17 years. The growth mixture modeling procedure [26] employed full information maximum likelihood (FIML) estimation with robust standard errors to address missing data. This method allows participants with incomplete data across repeated measures to be included and all available data to be used for estimating SU trajectories (n.b.: sample N at age 12 = 1355; age 13 = 1234; age 15 = 1446; age 17 = 1270; 92% of the sample had two or more valid SU assessments).

To address potential selective attrition, a comparison was made between the resulting subsample with valid SU data (N = 1593) and the participants without data during the key time points (N = 632; 28.4% of the cohort) with respect to socio-demographic variables collected at age 5 months. These variables included maternal and paternal age at birth, parental education, family income, family structure (intact or not), child sex, and the number of siblings. Using Cohen’s *h* as measure of effect size for the difference in proportion and *d* for the difference in mean (relative size of *d* or *h*: small effect ≥ 0.20; medium effect ≥ 0.50; large effect ≥ 0.80) [27], small but statistically significant differences were observed. Specifically, participants without data tended to have lower maternal (Cohen’s *h* = 0.11) and paternal education (*h* = 0.13), lower family income (Cohen’s *d* = 0.21), and a higher proportion of boys (*h* = 0.20). Further details and the strategy for dealing with selective attrition are provided in the Data Analysis section. Sample characteristics are available in Table 1.

#### Ethics

The protocol for the Québec Longitudinal Study of Child Development (QLSCD), which includes all data collection procedures, including questions relating to the health, safety, rights or privacy of research subjects, and the confidentiality of their personal information, has been approved by the Ethics Committee of the Institut de la statistique du Québec (ISQ). The ISQ is an agency of the Government of Québec that conducted the QLSCD. Informed written consent was obtained from participants and/or their parents separately for each wave of assessment. This study used QLSCD data obtained from a shared repository using only anonymized data and was conducted in accordance with the ethical standards set out in the 1964 Declaration of Helsinki and its subsequent amendment of 2013 (World Medical Association D-1964-01-2013).

### 2.2. Measures

#### 2.2.1. Substance Use

The frequency of alcohol, cannabis, and other illicit drug use was assessed through adolescents’ repeated self-reports at ages 12, 13, 15, and 17 years. The assessment included three items from the Personal Experience Screening Questionnaire (PESQ) [28,29]: “In the last 12 months, how many times did you use alcohol? How many times did you use cannabis? How many times did you use other illicit drugs such as cocaine, stimulants, speed, amphetamines, tranquilizers, inhalants, heroin, opiates, hallucinogens, psychedelics, or other narcotics or hard drugs?” Each response was coded on a seven-point scale: 0 = Never, 1 = Just once to try, 2 = Less than once a month, 3 = About once a month, 4 = Weekends or once/twice a week, 5 = Three times or more a week but not daily, 6 = Daily.

#### 2.2.2. Risk Factors

The measurement strategy for risk factors was designed to leverage the diverse data available in the study, incorporating individual, family, and social risk indicators from the pre-adolescent period. Risk factors were assessed at ages 10 and 12 years through reports from parents, teachers, and the children themselves. Overall, 4.3% of missing data points were found in the risk factor variables, a proportion (≤5%) generally considered inconsequential [30]. The inclusion of multiple informants aimed to enhance the validity of the risk factor measures [31], while the use of data from both 10 and 12 years was intended to increase the representativeness and reliability of the preadolescent indicators [32]. Unless otherwise noted, continuous or discrete measures from both ages were aggregated, and the coefficients reported represent the average of these statistics across the two time points. Details on the specific items used, as well as the construction of variables and scales, are available elsewhere [11].

The individual risk factors considered in this study included sex and both internalizing and externalizing problems. Internalizing problems included Anxiety (e.g., “fearful or anxious”; “worried”; average α = 0.73), Emotional problems (e.g., “seemed unhappy/sad”; “has difficulty having fun”; α = 0.73) based on teacher and child reports, and Depressive symptoms (e.g., “I always felt alone”; “Nobody really loved me”; α = 0.75) based on child report. Scores for each sub-scale were standardized, averaged by informant and assessment age, and then combined into a global preadolescent internalizing indicator. Externalizing problems included measures of Hyperactivity–Impulsivity (e.g., “could not sit still”; “difficulty taking turns”; average α = 0.75), Inattention (e.g., “easily distracted”; “inattentive”; α = 0.80), Opposition (e.g., “rebellious or refused to obey”; “got angry quickly”; α = 0.72), and Conduct problems (e.g., “stole things”; “got into a fight”; α = 0.75), based on teacher and child reports. As with internalizing problems, sub-scale scores were standardized, averaged by informant and assessment age, and then combined into a global preadolescent externalizing indicator.

Familial risk factors were assessed based on family adversity and appropriate parenting, measured at ages 10 and 12. Family adversity was defined using several socio-demographic indicators: maternal age at the birth of her first child, both maternal and paternal ages at the birth of the target child, and family structure (coded as “intact” if the child lived with both biological parents, or “non-intact” if the child did not). Using these baseline variables (Table 1), one adversity point was assigned to each of the following: (a) maternal age at or below the 30th percentile of the sample, (b) paternal age at or below the 30th percentile of the sample, and (c) a “non-intact” family structure. These adversity points were averaged to create a Family Adversity Index, which ranged from 0 to 1.

Appropriate parenting was evaluated using maternal ratings of both positive and coercive parenting practices, adapted from [33]. Positive parenting practices included behaviors such as “talking or playing with the child” and “doing sports or hobbies together.” Coercive practices were measured by items like “grasping firmly” and “raising your voice, scolding, or yelling at the child,” with the responses reverse-coded. Both sets of practices were rated on a 6-point scale (ranging from “never” (0) to “many times per day” (6)), and scores were summed to produce a total score. These scores were then standardized at each time point and averaged to create an overall preadolescent indicator of appropriate parenting (α = 0.64).

Social risk factors included household socioeconomic status (SES) and association with deviant peers. Household SES was a standardized composite based on parental occupational prestige, education level, and income [34]. Association with deviant peers was measured using two items: one based on children’s self-reports (“I hang out with a group of peers who do bad things”) and the other based on maternal reports (“my child hangs out with disruptive children”). Both items were coded as “yes” (1) or “no” (0), and the responses from both informants and assessment time points were summed to create a 4-point index. This index reflected both the presence of association with deviant peers and the severity of this association, considering whether both informants reported the behavior and whether it was reported at both time points.

### 2.3. Age-17 Outcomes

Substance Use-Related Outcomes included four self-reported indicators of substance use assessed for the past 12 months [35]. Daily smoking was coded as a binary variable (0 = no, 1 = yes). To measure alcohol abuse, participants reported the number of occasions they consumed five or more drinks in one sitting over the past year. Substance use variety was calculated by summing the number of different substances used in the same period, yielding a 7-point ordinal scale (Ordinal α = 0.70). Substance use-related problems were assessed through self-reported issues attributed to substance use, including psychological difficulties, harm to family or peer relationships, school problems, delinquent behaviors, reduced effectiveness of substances, and consulting for substance use concerns. Each problem was coded as 0 (no) or 1 (yes), and the sum produced a 0–7 scale (Ordinal α = 0.83).

Psychosocial maladjustment was evaluated using four self-reported indicators [33], all referring to the past year. Anxiety Symptoms: Nine items measured anxiety (e.g., “I have been too fearful or nervous”, “I worried about my health”), each coded as 0 (no), 1 (sometimes), or 2 (often). Scores were summed to create an 18-point scale (α = 0.79). Depression Symptoms: Eight items assessed depression symptoms (e.g., “I felt sad and unhappy”; “I lacked energy or felt tired”), with the same coding as above. This resulted in a 16-point scale (α = 0.85). Conduct Problems: Fifteen items evaluated conduct-related issues (e.g., “I have stolen money”; “I intentionally destroyed property”). Items were coded 0 (no), 1 (sometimes), or 2 (often), producing a 32-point scale (α = 0.80). Problems with the justice system: Five items captured interactions with the legal system (e.g., “I was arrested by the police”; “I was questioned by police”), coded 0 (no), 1 (sometimes), or 2 (often). This resulted in a 10-point scale (α = 0.79).

### 2.4. Data Analysis

To address differential attrition, inverse probability weighting was applied [36]. A logistic regression model was used to estimate the probability that participants would have valid data from ages 10 to 17 based on variables that differed between retained participants and those lost at follow-up. Each observation was then weighted by the inverse of its estimated probability. This approach has been shown to reduce bias associated with differential attrition in longitudinal studies [36].

A series of preliminary bivariate analyses were conducted to test whether the associations between each risk factor and at least one age-17 outcome met a minimal significance criterion (*p* < 0.25) to be included as control in the multivariate analyses [37]. All predictors met this criterion (Table 2).

Multicollinearity among risk factors was evaluated and found to be within acceptable limits [38]. Of the 21 bivariate associations examined (Table 3), only two correlation coefficients (0.52 and 0.61) exceeded 0.4 but remained below the critical threshold of 0.70, accounting for less than 38% of the shared variance in the correlated measures.

A Latent Class Three-Parallel Process Growth Mixture Modeling (PP-GMM) procedure was performed to identify patterns of concurrent substance use (SU) involving three types of substances over time. This approach integrates variable-centered and person-centered analyses. Analyses were conducted using full information maximum likelihood estimation with robust standard errors (MLR) in Mplus (version 8.11) [26]. MLR accommodates ordinal or count data, handles skewed and non-normal distributions, and provides unbiased estimates even in the presence of model or distributional misspecifications. The PP-GMM procedure adhered to established guidelines [39]. A detailed description of the PP-GMM analysis is available in earlier work identifying SU/PSU trajectories and their preadolescent risk factors [11].

Adolescent SU/PSU trajectory classes were examined in relation to age-17 outcomes while controlling for preadolescent risk factors. Each user class was compared to a non-user reference class. Probabilities of assignment to the most likely trajectory class were used as case weights to account for classification uncertainty [40,41]. Multivariate analyses were conducted using a Generalized Linear Model (GLM) framework [42], which accommodates normal, binary, or categorical data to compare trajectory classes across outcomes with different coding schemes. For each outcome, a baseline model including only covariates (risk factors) was first tested, followed by a full model incorporating both trajectory classes and covariates. Model fit improvement was evaluated using the likelihood ratio test (LRT). Because the analysis was repeated for the eight outcomes examined, False discovery rate (FDR) control was used (FDR = 5%) to adjust for multiple testing [43]. Finally, in order to provide an appraisal of the magnitude of the observed associations, Cohen’s d (for differences between means) or h (for differences between proportions) [27] estimates of effect size (corrected for the comparison of classes with different sample size) [44] were computed for each period.

### 2.5. Data Availability

The data used in this study were obtained from the final master file (1998–2015) of the Québec Longitudinal Study of Child Development (QLSCD), © Gouvernement du Québec, conducted by the Institut de la statistique du Québec (ISQ). As stipulated in clauses 10 and 11 of the Institut de la statistique’s Québec Act (Canada), access to the data is restricted to the parties identified in the partnership agreement signed to ensure the conduct of the study and which describes the author’s right. In the QLSCD cohort, the participants only consented to share their data with the study’s financial partners and affiliated researchers and their collaborators. Those partners and researchers only have access to the data after signing a data sharing agreement. Requests to access these data can be directed to the Institut de la statistique du Québec’s Research Data Access Services—Home (www.quebec.ca). For more information, contact Marc-Antoine Côté-Marcil (SAD@stat.gouv.qc.ca). See also: http://www.jesuisjeserai.stat.gouv.qc.ca/informations_chercheurs/acces_an.html.

## 3. Results

### 3.1. Developmental Patterns of PSU

**Figure 1:**
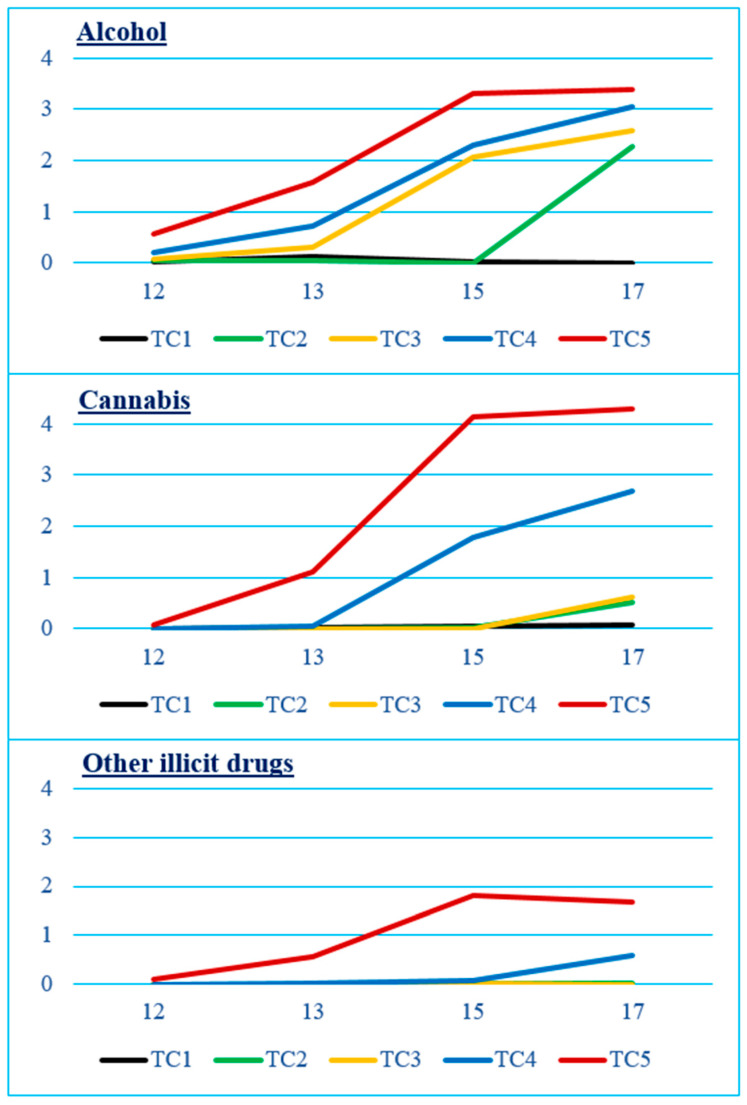
Frequency of alcohol (AL), cannabis (CA), and other illicit drug (OD) use for each trajectory-class (TC) of users from age 12 to 17 years. Trajectory-classes (TC) are: TC1, Non-users (N = 204, 12.8% sample); TC2, Later-onset/experimental (exp.) AL and CA (N = 362, 22.7%); TC3, Increasing AL and later-onset/exp.CA (N = 597, 37.5%); TC4, Increasing AL + CA and later-onset/exp.OD (N = 337, 21.2%); TC5, Early-onset increasing AL + CA + OD (N = 93, 5.8%). Frequency of use in the past 12 months: 0—Never, 1—Just once to try, 2—Less than once a month, 3—About once a month, 4—Weekends or once–twice a week, 5—Three times or more a week, but not every day, 6—Daily. Adapted from [11], © 2024 American Psychological Association.

The final model identified five distinct trajectory classes describing adolescents’ SU/PSU [11], as illustrated in Figure 1. The first class (TCL1 in Figure 1) consisted of non-users throughout adolescence (N = 204, 12.8% of the sample). The second class (TCL2), comprising later-onset/experimental alcohol (AL) and cannabis (CA) users (N = 362, 22.7%), demonstrated initial use beginning after age 15. The third class (TCL3), increasing AL and later-onset/experimental CA users (N = 597, 37.5%), exhibited a gradual increase in AL use starting from age 13 and CA use beginning after age 15, reaching a limited level of use thereafter. The fourth class (TCL4) showed a pattern of increasing AL and CA use, with later-onset/experimental OD use (N = 337, 21.2%). This class experienced a steady rise in AL and CA used to moderate levels starting at age 13, with OD use beginning after age 15. The fifth class (TCL5), early-onset increasing AL, CA, and OD users (N = 93, 5.8%), included adolescents who initiated AL use by age 12 and CA and OD use by age 13. They demonstrated a sharp increase in AL and CA use up to age 15, followed by a moderate increase in OD use, with all three substances maintained at a similar level of use throughout age 17.

### 3.2. Association Between Adolescent SU/PSU Trajectory-Classes and Age-17 Outcomes

For all outcomes, the full model that included both trajectory classes and covariates provided a better fit than the baseline model, which included only covariates (risk factors), as demonstrated by the results of the Likelihood Ratio Test (LRT) comparing the two models. The associations between SU/PSU trajectory classes and substance-related outcomes are detailed in Table 4. Adolescents in the Increasing AL + CA and later-onset/experimental OD and Early-onset increasing AL + CA + OD PSU classes reported higher levels of difficulties compared to their peers in the non-users reference class. These two classes of polysubstance users were also more likely to report the four substance use-related outcomes than adolescents in the Later-onset/experimental AL and CA and Increasing AL and later-onset/experimental CA classes. Regarding the magnitude of these associations, effect sizes for early-onset increasing AL + CA + OD users (average: 2.31; range: 1.18–4.53) were approximately twice as large as those for Increasing AL + CA and later-onset/experimental OD users (average: 1.13; range: 0.50–2.47), consistent with the higher levels observed for the former in three of the four outcomes (excluding the frequency of 5+ drinks). The two lower user classes reported a higher frequency of alcohol abuse (i.e., having five or more drinks on a single occasion) and substance-related problems than the Non-users reference class. Adolescents in the Increasing AL and later-onset/experimental CA class also reported a higher frequency of alcohol abuse than those in the Later-onset/experimental AL and CA class, although the magnitude of these associations (average: 0.59; range: 0.00–2.36) was considerably lower than those observed in the two higher PSU classes.

Regarding psychosocial outcomes (Table 5), participants in the two higher PSU classes were more likely to report maladjustment across all indicators compared to their peers in the reference class. The 95% confidence intervals (CIs) suggested larger effect sizes for Early-onset increasing AL + CA + OD users compared to Increasing AL + CA and later-onset/experimental OD users for conduct problems and problems with the justice system.

Participants in the two higher PSU classes also reported elevated levels of all psychosocial outcomes relative to the two lower user classes. However, the latter still displayed higher maladjustment than the Non-users reference class for three out of four measures, with the exception of Problems with the justice system. Overall, for both higher (i.e., TCL4 and TCL5) and lower (TCL2 and TCL3) SU/PSU classes, the effect sizes for the associations with psychosocial outcomes (average: 0.79 and 0.22, respectively) were smaller than those shown for substance-related outcomes (average: 1.72 and 0.59, respectively).

## 4. Discussion

The present study examined age-17 substance-related and other psychosocial outcomes associated with adolescent developmental trajectories of substance use or polysubstance use (SU/PSU) in a population-representative cohort. Specifically, we investigated the contribution of adolescent SU/PSU—i.e., alcohol (AL), cannabis (CA), or other illicit drugs (ODs)—to these outcomes while controlling for preadolescent risk factors linked to SU/PSU trajectories. As expected, adolescents with a pattern of higher-risk PSU (i.e., Increasing AL + CA and later-onset/exp.OD and Early-onset increasing AL + CA + OD users) showed the most negative outcomes for all substance-related and psychosocial measures in comparison to low/non-users as well as to lower-risk SU/PSU trajectory-classes (i.e., Later-onset/experimental AL and CA and Increasing AL and later-onset/exp.CA). Moreover, adolescents in the Early-onset increasing AL + CA + OD class stood out by showing higher levels of three out of the four substance-related outcomes (except the frequency of 5+ drinks) and more conduct problems and problems with the justice system compared to their Increasing AL + CA and later-onset/exp.OD peers. Our hypotheses were also supported by the fact that participants in lower-risk SU/PSU trajectory-classes showed higher maladjustment than low/non-users for most examined outcomes, but to a lesser extent than their peers showing higher-risk PSU patterns.

Together, the results demonstrate a dose–response relationship between longitudinal patterns of SU/PSU during adolescence and outcomes at age 17. These findings are consistent with prior research with this sample showing that the type, number, and severity of the same preadolescent risk factors portend the severity of adolescent user trajectories used in this report [11]. Notably, even lower-risk adolescent SU/PSU is associated with negative outcomes, aligning with previous studies [12,13,14]. The risk of adverse outcomes increases with earlier initiation and more frequent use of multiple substances (i.e., more than two) during adolescence. These results highlight the critical role of SU/PSU patterns from early to late adolescence in predicting adverse outcomes, independent of preadolescent individual, familial, and social risk factors.

The analytical strategy employed enabled the identification of distinct adolescent risk classes based on their consumption profiles over time relative to peers. The mean SU levels for the highest-risk class across all substances did not exceed 4.3 (corresponding to the ‘4-weekends or once–twice a week’ category) in the present study. Nevertheless, the findings suggest that this level of use in a polysubstance context represents a significant risk for substance-related and psychosocial problems by age 17. The severity of usage patterns is determined by factors such as onset, intensity, and the number of substances used. Furthermore, the majority of adolescent substance users in the population sample reported using two or more substances, consistent with earlier studies [6,7]. These findings strongly support the need to move beyond studying single substances to investigating multiple substances for a comprehensive picture of adolescent substance involvement [3,6,7,8,10,19].

Importantly, the dose–response relationship between adolescent SU/PSU patterns and maladjustment at age 17 was observed for both substance-related and psychosocial outcomes. The analyses accounted for preadolescent risk factors previously linked to adolescent patterns of SU/PSU (male sex, externalizing and internalizing problems, family adversity, parenting, socioeconomic status, and affiliation with deviant peers; for details, see [11]), which are also factors typically associated with the psychosocial outcomes examined in this study [22,23]. Thus, above and beyond the influence of common risk factors, the relative severity of SU/PSU patterns between ages 12 and 17 is significant, primarily regarding substance-related outcomes but also to psychosocial maladjustment at the end of this period. The results indicate that most youths initiated SU or PSU after preadolescence, suggesting that this latter period presents a critical window of opportunity to identify high-risk individuals and implement intensive, selective, or indicated preventive interventions [45,46]. Such interventions could help prevent or delay the onset of SU, curb the escalation to PSU, and mitigate subsequent substance-related and psychosocial issues [45,46,47,48].

The present findings do not rule out the possibility that preadolescent risk factors associated with adolescent SU and PSU may also give rise to new, unmeasured, concurrent risk factors during adolescence. These emergent risk factors (e.g., association with substance using peers), however, would be the continuation of earlier risk factors and some of them could become entangled with SU/PSU patterns [22,23,49]. Such factors may nevertheless contribute to the persistence of SU/PSU and lead to adverse outcomes on their own, as suggested by residual associations between many of these factors and the outcomes at age 17 [22,48,50].

The differential associations observed between preadolescent risk factors and patterns of adolescent SU/PSU [11], as well as the links with outcomes at age 17, align with the developmental model of SU [20,21]. This model integrates individual (e.g., externalizing behaviors), familial (e.g., inadequate parenting), and social (e.g., low socioeconomic status, deviant peer influences) risk factors to describe the sequential, multilevel, additive, or transactional mechanisms that lead from early childhood to SU initiation and escalation, including PSU, and ultimately to substance use disorders and functional impairments. This developmental model has been contextualized within a broader framework of externalizing and internalizing symptom development [22,23]. According to this perspective, individual and environmental characteristics may also moderate the longitudinal relationships between adolescent SU/PSU (or preadolescent risk factors) and later outcomes. Individual differences, such as neurobiological vulnerability to substance effects, may influence the onset and progression of SU and PSU [47,51,52]. This vulnerability may be further exacerbated by SU/PSU during adolescence, potentially disrupting critical brain maturation processes [51,52]. Depending on the type and number of substances used, such disruptions may result in a range of cognitive and behavioral consequences, including impairments in attention, memory, learning, impulsivity, and behavior regulation, which could have long-term implications for academic, social, and mental health outcomes [8,51,53].

### 4.1. Implication for Future Studies and Practice

The results of this study highlight important aspects that should be considered in future studies. First, research on adolescent SU should be based on longitudinal studies that include repeated assessments of multiple substances to provide a thorough and nuanced understanding of this serious and enduring phenomenon [6,7,8]. Second, studies of adolescent SU outcomes should adopt a broad developmental perspective that encompasses antecedent risk factors to disentangle the specificity of the influences involved [22,23,24]. Third, these studies should also examine the contributing factors that are concomitant with the development of adolescent SU, as well as their underlying mechanisms during this critical period for brain development [47,48]. Such a broad perspective should allow for better screening of preadolescents at risk of SU, while informing practitioners about key aspects that could be targeted by universal, selective, and indicated preventive interventions across developmental periods [45,46].

### 4.2. Strengths and Limitations

The present study has several notable strengths, including the use of a large population-based cohort and multiple assessments of different substances to estimate adolescents’ developmental patterns of substance use (SU) and polysubstance use (PSU). Employing an integrated variable- and person-centered approach provided a robust account of adolescents’ developmental trajectories compared to their peers, avoiding the limitations of cross-sectional and substance-specific investigations. Additionally, controlling for preadolescent individual, familial, and social risk factors allowed for a more accurate assessment of the potential influence of adolescent SU/PSU patterns on age-17 outcomes.

However, several limitations should be acknowledged. First, the assessment of adolescents’ SU/PSU relied on self-reports, which may have been influenced by self-presentation concerns or recall bias. Despite this limitation, self-reports remain the most practical method for large, population-based studies and are widely used in general population surveys [1]. Notably, prior research has demonstrated that adolescents aged 12 to 17 tend to report recent substance use accurately [54]. Second, the measures used in this study did not specify the exact type of PSU beyond detailing the substances and their frequency of use over the past year. A shorter reference period or an assessment of simultaneous substance use could potentially yield a smaller proportion of polysubstance users, with stronger associations with risk factors and outcomes. However, the chosen approach likely captured a broader range of users, including lower-frequency users who are also at risk [12,13,14], making it more efficient for population-based sampling.

Third, the age-17 outcomes were also based on self-reports, which could have inflated the associations with SU/PSU due to shared method variance. Fourth, it is important to emphasize the associative, rather than causal, nature of the findings. This limitation is particularly relevant because preadolescent risk factor measures were partially based on age-12 reports, assessed concurrently with the initial SU/PSU measures, while outcome measures were assessed concurrently with the final SU/PSU assessments. Finally, the study sample consisted predominantly of French-speaking adolescents from Quebec, Canada, which may limit the generalizability of the findings. Replications in more diverse populations are needed to confirm these results.

### 4.3. Conclusions

The present study highlights the dose-response association between adolescent SU/PSU patterns and age-17 substance-related and psychosocial outcomes, independent of preadolescent risk factors. These findings underscore the importance of longitudinal investigations that consider multiple substances to comprehensively capture adolescents’ substance use involvement. Future research should build on this approach to better understand both the early factors that contribute to different adolescent SU/PSU patterns and the concurrent factors that sustain SU/PSU and its association with later adjustment difficulties. This deeper understanding will inform the development of timely, tailored intervention programs to address the specific needs of children and adolescents at risk.

## Figures and Tables

**Table 1:** Sample characteristics and risk factor variables at age 10–12 years.

Measures and Indicators	Mean (SD) or %
Sample characteristics	
Race: White of European ancestry (%)	92.0
Immigrant status: Mother/Father (%)	8.5/9.9
French/English as first language: Mother (%)	85.2/6.6
French/English as first language: Father (%)	83.4/7.3
Language spoken by parents at home: French or English/Other (%)	97.7/2.3
Mother/No High school diploma (%)	20.4
Father/No High school diploma (%)	24.6
Maternal age at birth of first child	26.7 (9.1)
Maternal age at child’s birth	29.3 (5.2)
Paternal age at child’s birth	32.6 (8.1)
Number of siblings	1.4 (0.88)
Non-intact family (%)	36.7
Household income ($)	72,800 (37,200)
Risk factors ^1^	
Individual	
Sex (%): male; female	48.4; 51.6
Internalizing	0.33 (0.35)
Externalizing	0.32 (0.32)
Familial	
Family adversity	0.31 (0.26)
Appropriate parenting	0.58 (0.16)
Social	
Household SES	0.00 (1.00)
Association with deviant peers	0.22 (0.23)

^1^ SES is a standardized indicator. Except for Sex, other risk factor variables (mean, (SD) are shown) are converted into 0 to 1 indicators in the present table to enable comparison between differently coded variables. Underlining denotes risk factor categories.

**Table 2:** Association coefficients between risk factor variables and age-17 outcomes.

Risk Factors/Control Variables	Substance-Related Outcomes				Psychosocial Adjustment Outcomes			
	Daily Smoker ^a^	Frequency of 5+ Drinks on Same Occasion	Variety of Substances Used	Problems related to SU	Anxiety Symptoms	Depression Symptoms	Conduct Problems	Problems with the Justice System
Male sex ^a^	0.03	0.00	0.02	0.05 *	−0.36 ***	−0.32 ***	0.02	0.09 ***
Internalizing problems	0.11 ***	−0.10 ***	0.02	0.08 **	0.22 ***	0.26 ***	0.12 ***	0.09***
Externalizing problems	0.18 ***	0.10 ***	0.15 ***	0.21 ***	0.08 **	0.09 ***	0.26 ***	0.22 ***
Family adversity	0.07 **	0.04	0.11 ***	0.09 ***	0.03	0.07 **	0.14 ***	0.15 ***
Appropriate parenting	−0.19 ***	−0.03	0.02	0.00	−0.01	0.01	−0.02	−0.04
SES	−0.15 ***	0.04	−0.07 **	−0.04	−0.01	−0.02	−0.08 **	−0.12 ***
Deviant peers	0.11 ***	0.15 ***	0.15 ***	0.21 ***	0.02	0.02	0.19 ***	0.14 ***

Spearman *rho*, *Phi*, or *Eta* coefficients were computed depending on the nature of the variables involved in the associations. ^a^: Binary variable. *: *p* < 0.05, **: *p* < 0.01, ***: *p* < 0.001. Underlining denotes outcome categories.

**Table 3:** Association coefficients among risk factor variables.

Risk Factors	1	2	3	4	5	6	7
Individual							
1-Male sex ^a^		0.13 ***	0.30 ***	0.01	0.09 ***	0.01	0.17 ***
2-Internalizing			0.61 ***	0.14 ***	0.01	−0.17 ***	0.21 ***
3-Externalizing				0.16 ***	0.05	−0.16 ***	0.52 ***
Familial							
4-Family adversity					−0.02	−0.38 ***	0.09 **
5-Appropriate parenting						0.12 ***	0.01
Social							
6-Household SES							−0.08 **
7-Association with deviant peers							

Spearman *rho*, *Phi*, or *Eta* coefficients were computed depending on the nature of the variables involved in the associations. ^a^: Binary variable. **: *p* < 0.01, ***: *p* < 0.001.

**Table 4:** Association between adolescent trajectory-classes of SU and substance-related outcomes at age 17.

Trajectory-Classes and Control Variables	Daily Smoker % *p* *h* 95% CI		Frequency of 5+ Drinks on Same Occasion Mean (SD) *p* *d* 95% CI		Variety of Substances Used Mean (SD) *p* *d* 95% CI		Problems Related to Substance Use Mean (SD) *p* *d* 95% CI	
(1) Non-users: Reference— class (N = 204, 12.8%)	1.2		0.00 (0.00)		0.00 (.00)		0.01 (0.07)	
(2) Later-onset/exp. AL and CA (N = 362, 22.7%)	2.2_4,5_ 0.938 0.08 [−0.10–0.25]		1.73 (1.73)_3,4,5_ < 0.001 1.25 [1.06–1.44]		0.03 (.35)_3–5_ < 0.001 0.11 [−0.07–0.28]		0.17 (.65)_4,5_ 0.001 0.31 [0.13–0.48]	
(3) Increasing AL and later- onset/exp. CA (N = 597, 37.5%)	1.4_4,5_ 0.653 0.02 [−0.14–0.18]		2.36 (1.88)_2,4,5_ < 0.001 1.45 [1.28–1.63]		0.00 (.00)_2,4,5_ 1.00		0.16 (.58)_4,5_ 0.001 0.30 [0.14–0.46]	
(4) Increasing AL + CA and later- onset-exp. OD (N = 337, 21.2%)	12.3_2,3,5_ < 0.001 0.50 [0.25–0.60]		3.43 (1.76)_2,3_ < 0.001 2.47 [2.24–2.70]		0.43 (.75)_2,3,5_ < 0.001 0.73 [0.55–0.91]		0.94 (1.43)_2,3,5_ < 0.001 0.82 [0.64–1.00]	
(5) Early-onset increasing AL + CA + OD (N = 93, 5.8%)	41.6_2–4_ < 0.001 1.39 [1.12–1.66]		3.92 (1.55)_2,3_ < 0.001 4.53 [4.09–4.97]		1.31 (1.34)_2–4_ < 0.001 1.75 [1.47–2.03]		2.46 (2.46)_2–4_ < 0.001 1.78 [1.50–2.07]	
Trajectory-classes (*X*^2^ (df) *p*)	99.23 (4) < 0.0001		58.25 (4) < 0.0001		109.79 (4) < 0.0001		313.30 (4) < 0.0001	
Control variables (*Beta*; *p*)								
Male sex	0.298	0.274	0.013	0.849	−0.051	0.773	0.175	0.147
Internalizing problems	0.150	0.291	−0.111	0.006	−0.075	0.446	0.023	0.736
Externalizing problems	0.498	0.003	0.082	0.073	0.182	0.094	0.198	0.010
Family adversity	0.227	0.048	−0.016	0.633	−0.036	0.651	−0.004	0.948
Appropriate parenting	−0.147	0.247	−0.008	0.800	−0.228	0.007	0.079	0.173
SES	−0.300	0.058	0.040	0.285	−0.297	0.004	0.108	0.112
Deviant peers	−0.271	0.052	0.060	0.105	0.074	0.402	0.098	0.103
Model fit (*X*^2^ (df) *p*)	185.50 (11) < 0.00001		559.78 (11) < 0.00001		575.23 (11) < 0.00001		593.46 (11) < 0.00001	
LRT (*X*^2^ (df) *p*)	122.236 (4) < 0.00001		508.756 (4) < 0.00001		480.608 (4) < 0.00001		457.311 (4) < 0.00001	

The *p* indicated is for model comparing user trajectory-classes with the Non-users reference class. False Discovery Rate control across the eight comparisons of Table 1 and Table 2 showed that results indicated as significant remained within the corrected significance range (FDR = 5%). Therefore, the original *p* are shown. Significant comparison between user classes and the corresponding class in subscript number, at *p* < 0.05 after FDR correction was applied. Model fit *X*^2^ is for full model displayed. Likelihood ratio test (LRT) between baseline model (only covariates) and full model (trajectory classes and covariates). Measures of effect size *h*, *d*: Cohen’s *h* for the difference in proportion and *d* for the difference in mean with the reference class. Relative size of Cohen’s *h*, *d*: small effect ≥ 0.20; medium effect ≥ 0.50; large effect ≥ 0.80 [27].

**Table 5:** Association between adolescent trajectory-classes of SU and psychosocial adjustment outcomes at age 17.

Trajectory-Classes and Control Variables	Anxiety Symptoms Mean (SD) *p* *d* 95% CI		Depression Symptoms Mean (SD) *p* *d* 95% CI		Conduct Problems Mean (SD) *p* *d* 95% CI		Problems with the Justice System Mean (SD) *p* *d* 95% CI	
(1) Non-users: Reference— class (N = 204, 12.8%)	3.81 (2.08)		3.09 (2.07)		0.35 (0.77)		0.15 (0.73)	
(2) Later-onset/exp. AL and CA (N = 362, 22.7%)	4.30 (2.17)_4,5_ 0.004 0.23 [0.06–0.40]		3.91 (2.30)_4,5_ < 0.001 0.37 [0.20–0.54]		0.64 (.88)_4,5_ 0.001 0.34 [0.17–0.52]		0.18 (.70)_3,4,5_ 0.586 0.04 [−0.13–0.21]	
(3) Incr. AL later-onset-exp. CA (N = 597, 37.5%)	4.25 (2.12)_4,5_ 0.011 0.21 [0.05–0.37]		3.76 (2.21)_4,5_ < 0.001 0.31 [0.15–0.47]		0.58 (.81)_4,5_ 0.008 0.29 [0.13–0.45]		0.09 (.46)_2,4,5_ 0.019 −0.11 [−0.27–0.05]	
(4) Incr. AL + CA and later- onset/exp. OD (N = 337,21.2%)	4.97 (2.27)_2,3_ < 0.001 0.53 [0.35–0.70]		4.37 (2.40)_2,3_ < 0.001 0.56 [0.38–0.74]		1.24 (1.10)_2–3_ < 0.001 0.90 [0.72–1.08]		0.38 (1.04)_2,3,5_ < 0.001 0.25 [0.07–0.42]	
(5) Early-onset incr. AL + CA + OD (N = 93, 5.8%)	5.20 (2.09)_2,3_ < 0.001 0.67 [0.42–0.92]		4.93 (2.60)_2,3_ < 0.001 0.82 [0.56–1.07]		1.99 (1.39)_2–3_ < 0.001 1.63 [1.35–1.91]		1.31 (1.83)_2–4_ < 0.001 0.98 [0.72–1.23]	
Trajectory-classes (*X*^2^ (df) *p*)	47.56 (4) < 0.0001		67.30 (4) < 0.0001		102.06 (4) < 0.0001		124.93 (4) < 0.0001	
Control variables (*Beta*; *p*)								
Male sex	−1.603	<0.001	−1.507	<0.001	−0.044	0.554	0.443	0.001
Internalizing problems	0.446	<0.001	0.613	<0.001	0.044	0.345	−0.014	0.848
Externalizing problems	0.218	0.002	0.156	0.032	0.255	<0.001	0.534	<0.001
Family adversity	−0.035	0.485	0.059	0.319	0.047	0.313	0.198	0.001
Appropriate parenting	0.004	0.942	0.040	0.617	−0.049	0.617	−0.104	0.125
SES	0.103	0.067	0.114	0.101	−0.011	0.575	−0.189	0.013
Deviant peers	−0.067	0.246	−0.101	0.102	0.023	0.599	−0.071	0.313
Model fit (*X*^2^ (df) *p*)	369.30 (11) < 0.00001		383.48 (11) < 0.00001		200.24 (11) < 0.00001		345.96 (11) < 0.00001	
LRT (*X*^2^ (df) *p*)	46.862 (4) < 0.00001		65.908 (4) < 0.00001		105.400 (4) < 0.00001		135.746 (4) < 0.00001	

The *p* indicated is for model comparing user trajectory-classes with the non-users reference class. False Discovery Rate control across the eight comparisons of Table 1 and Table 2 showed that results indicated as significant remained within the corrected significance range (FDR = 5%). Therefore, the original *p* are shown. Significant comparison between user classes and the corresponding class in subscript number, at *p* < 0.05 after FDR correction was applied. Model fit *X*^2^ is for full model displayed. Likelihood ratio test (LRT) between baseline model (only covariates) and full model displayed (trajectory classes and covariates). Measure of effect size: Cohen’s *d* for the difference in mean with the reference class. Relative size of Cohen’s *d*: small effect ≥ 0.20; medium effect ≥ 0.50; large effect ≥ 0.80 [27].

## Data Availability

The data used in this study were obtained from the final master file (1998–2015) of the Québec Longitudinal Study of Child Development (QLSCD), © Gouvernement du Québec, conducted by the Institut de la statistique du Québec (ISQ). As stipulated in the clauses 10 and 11 of the Institut de la statistique’s Québec Act (Canada), the access to the data is restricted to the parties identified in the partnership agreement signed to ensure the conduct of the study and which describes the author’s right. In the QLSCD cohort, the participants only consented to share their data with the study’s financial partners and affiliated researchers and their collaborators. Those partners and researchers only have access to the data after signing a data sharing agreement. Requests to access these data can be directed to the Institut de la statistique du Québec’s Research Data Access Services—Home (www.quebec.ca). For more information, contact Marc-Antoine Côté-Marcil (SAD@stat.gouv.qc.ca).
